# Exploring Factors Influencing Immunization Utilization in Nigeria—A Mixed Methods Study

**DOI:** 10.3389/fpubh.2019.00392

**Published:** 2019-12-20

**Authors:** Ngozi N. Akwataghibe, Elijah A. Ogunsola, Jacqueline E. W. Broerse, Oluwafemi A. Popoola, Adanna I. Agbo, Marjolein A. Dieleman

**Affiliations:** ^1^Global Health Department, Royal Tropical Institute, Amsterdam, Netherlands; ^2^Athena Institute, Faculty of Science, Vrije Universiteit Amsterdam, Amsterdam, Netherlands; ^3^Ogun State Primary Health Care Development Board, Abeokuta, Nigeria; ^4^Amsterdam Public Health Research Institute, Amsterdam, Netherlands; ^5^Department of Community Medicine, College of Medicine, University of Ibadan, Ibadan, Nigeria; ^6^Nursing Department, School of Community Health and Policy, Morgan State University, Baltimore, MD, United States

**Keywords:** immunization, utilization, vaccine hesitancy, community links, health services, household decision-making

## Abstract

**Background:** In 2005, Nigeria adopted the Reach Every Ward strategy to improve vaccination coverage for children, 0–23 months. By 2015, Ogun state had full coverage in 12 of its 20 local government areas but eight had pockets of unimmunized children, with the highest burden (37%) in Remo-North. This study aimed to identify factors in Remo-North influencing the use of immunization services, in order to inform intervention approaches to tackle barriers to immunization utilization.

**Methods:** We carried out a cross-sectional study using mixed methods including a survey of caregivers of 215 children, 25 semi-structured interviews with stakeholders involved in immunization service delivery and 16 focus group discussions with community men and women (*n* = 98). Two wards (Ilara and Ipara) were purposively chosen for the study. Data was analyzed using the SAGE Working Group Vaccine Hesitancy model.

**Results:** Only 56 children (32.6%) of the 172 children over 9 months of age had immunization cards available for inspection. Of these, 23 (59.6%) were fully immunized, noticeably higher in Ipara than Ilara. However, when immunization status was assessed by card and recall, 84.9% of the children were assessed as fully immunized. Caregivers in the more rural Ilara had less knowledge of vaccine schedules. The importance of all doses was recognized more by Ipara respondents (95.5%) than in Ilara (75.3%) (*p* < 0.05). Community links to immunization and household decision-making patterns influenced immunization use in both wards. Migrants and those living in hard-to-reach areas were disadvantaged in both wards. Health service factors like absence of delivery services, shortage of health workers, unavailability of vaccines at scheduled times, and indirect costs of immunization contributed to low utilization.

**Conclusion:** Immunization utilization was influenced by interlinked community and health services issues. Intervention approaches should ensure that communities' priorities are addressed, actors at both levels involved and strategies are adjusted to suit contexts.

## Introduction

Immunization is considered one of the most cost-effective health interventions, reducing under-five mortality (1). Global immunization coverage from 2010 to 2015 shows that at least 85% of children received three doses of diphtheria-pertussis-tetanus (DPT) vaccine (2). However, in 2015, the number of children without routine immunization (RI) was 19.4 million globally (2). The majority (75%) of non-immunized children live in 10 countries, including Nigeria (1).

Nigeria is the most densely populated country in Africa with an annual population growth rate of 2.83% (3) and is the second largest contributor to under–five mortality in the world (4). According to the 2013 National Demographic Health Survey (NDHS) (5), only 25% of children aged 12–23 months completed the prescribed course of RI. However, there are marked inequalities across geopolitical zones with immunization completion ranging from about 50% in the South-West and South-South to 27, 14, and 10% in the North-Central, North-East and North-West, respectively. Factors responsible for this poor performance (6, 7) include medical mistrust driven by socio-political factors (8, 9), weak health systems with poor patronage by clients, hostile attitudes of health workers, conflicts between competing programmes and between routine and supplemental immunization activities (10). Vaccines are usually procured by the Federal government with the support of donor organizations such as the Global Alliance for Vaccines and Immunization (GAVI). However, though these vaccines are supplied free to the states' primary health care development agencies, indirect costs of immunization due to logistics and illegal charges by health workers at the health facility level, limit vaccine availability to users (11).

Reaching Every Ward (REW), an approach developed in 2002 by the World Health Organization (WHO) and partners, provides a framework for strengthening national immunization programmes (12). In order to improve immunization coverage, Nigeria adopted the REW strategy in 2005. The REW strategy focuses on RI in health facilities and outreaches, including components such as improved access for under-served and hard-to-reach areas; support supervision; monitoring and use of data for action; community mobilization and improving community links with service delivery. These community linkages include the Ward Development Committee (WDC)-linked to primary health care including immunization at ward level; and the Social Mobilization Committee (SMC), focused specifically on immunization at local government level. The RI schedule is detailed in Box 1.

Box 1 **Routine immunization schedule.**

At Birth—BCG; OPV; HBV

6 Weeks—OPV; PENT A; PCV

10 Weeks—OPV; PENTA; PCV

14 Weeks—OPV; PENTA; PCV; IPV

9 Months—Yellow Fever Vaccine, Measles Vaccine and Vitamin A

BCG—Bacillus Calmette-Guérin

OPV—Oral Polio Vaccine

HBV—Hepatitis B Vaccine

*PENTA—Pentavalent Vaccine against Haemophilus influenzae type B*,

Diphtheria, Pertussis, Tetanus and Hepatitis B

PCV—Pneumococcal Conjugate vaccine

IPV—Inactivated polio vaccine

Since 2009, Ogun state in South-West Nigeria has recorded consistent increase in RI coverage in all its twenty Local Government Areas (LGAs) with coverage as high as 107%^1^. However, in 2015, there were still pockets of unimmunized children in eight LGAs, with a total of about 9,394 (16%) children unimmunized, and the highest proportion in Remo-North LGA (37%). The factors responsible for this trend in the eight LGAs were unknown.

This study aimed to identify factors influencing the use of immunization services in Remo-North, in order to inform intervention approaches to tackle barriers to immunization utilization.

## Methods

We carried out a cross-sectional study using mixed methods, comprising a household survey, focus group discussions and semi-structured interviews. We used the qualitative interviews to explain the results of the survey and to gain more insight into contextual factors. We used a convergent (concurrent) mixed methods design-the quantitative and qualitative data were collected in parallel, within the same time frame. Integration was carried out during data analysis and interpretation of results. Quantitative data provided a starting point for analysis, and qualitative data were then used to further explain the quantitative results. If areas of divergence emerged, we ascertained the cause of the disparity before drawing conclusions. For instance, we checked if the difference was caused by answers given by stakeholders due to hierarchy or social desirability; or due to researcher error such as framing of questions; or due to incorrect interpretation of results.

*Household survey of caregivers* responsible for the vaccination of at least one under-five child was conducted. Close-ended questionnaires were administered. Variables such as knowledge and utilization of immunization facilities, community links, and child's immunization details were collected. The primary study outcome was immunization completeness—assessed as three doses of Diphtheria, Pertussis, Tetanus (DPT)/Pentavalent vaccine as well as measles and yellow fever recorded as administered in an immunization card. The primary exposure variables were location, respondent's educational status, family wealth status, respondent's literacy, and employment status. Respondents were categorized as having no formal or only preschool education; or having primary, secondary or tertiary education. Respondent literacy was assessed by having them read a simple sentence “Remo North is a great place to live in.” Family wealth was assessed using standard wealth items of living condition, household amenities, and ownership of household assets from the National Demographic and Health Survey. Employment status responses were summarized as currently employed or unemployed.

*Focus Group Discussions (FGDs)* took place with community members. Participants were separated according to gender and age—women of child-bearing age and older women (above 40 years); young men and older men. FGDs provided insight into the expectations and needs of the communities regarding immunization, their perceptions of health and immunization services and existing community linkages to immunization service delivery.

*Semi-structured interviews (SSIs)* were carried out with frontline health workers, policy makers, local government implementers, religious and traditional leaders and community stakeholders in social mobilization structures (such as WDC and SMC) linked to immunization service delivery. These SSIs were used to gain insight into facilitators and barriers to improving immunization coverage; challenges in the implementation of immunization services; community collaborations for immunization service delivery; and stakeholders' perceptions of how immunization service delivery is matched to community needs.

Data collection was carried out in May 2016 by a team of two quantitative and two qualitative researchers and three research assistants. The researchers consisted of two men and two women—all with medical backgrounds. Three of them were academics. One male researcher was a policy maker from Ogun state and was not directly involved in the data analysis so as to reduce bias but he provided insight during interpretation of data. The two qualitative researchers were women. Fieldwork commenced with training and piloting of tools. Two trained research assistants functioned as coordinators in each ward, respectively, and a third coordinated all administrative and logistic processes.

### Research Setting

Remo-North LGA was purposively chosen for the study because it had the highest burden of unimmunized children in Ogun State. Ipara and Ilara, the best and worst performing wards were also purposively selected. For this study, we chose two focal wards from Remo-North LGA using the criteria of performance—determined by immunization coverage trends in the National Health Management Information System (NHMIS). Ipara and Ilara were the best and worst performing wards in Remo-North, respectively. We wanted to find out whether there were differences in the sites which could explain the outcome (immunization coverage). In 2015, immunization coverage in Ilara was remarkably low—with only 26% of children (compared to 78% in 2014) fully immunized. Ipara ward performed much better with 76% of children (compared to 69% in 2014) fully immunized, lagging slightly behind the National Programme on Immunization's acceptable minimum of 80%. From 2014 to 2015, immunization coverage improved across all the antigens in Ipara while in Ilara, coverage dropped precipitously across all antigens.

Both wards had similar socio-cultural contexts however there were a few differences. Ilara is located on the outskirts of Remo-North and is a farm settlement; it is more remote and rural than Ipara. Furthermore, the access road is bad, limiting commercial activities. Ipara is perceived to have more educated people than Ilara, is described as a “semi-rural” ward and has a more organized structure with numbered streets. The communal lifestyle of the Ilara people enables easy access to their king (Kabiyesi)—the prime traditional ruler of the ward. The “kings” are powerful figures in the wards and exert strong influence over the political, socio-cultural, and economic structures within their areas of jurisdiction. In Ipara, community members are relatively less dependent on their king though traditional protocols are observed.

### Sampling and Recruitment

The population of Ilara was 6,512 compared to 9,100 of Ipara (2017). The Yoruba tribal group are indigenous in the state and make up the majority of the population in both wards. According to the Ogun State Primary Health Care Development Board (SPHCDB), Department of Research and Statistics (2019), Non-indigenous groups make up about 10% (600 and 974 people in Ilara and Ipara, respectively) of the population. Non-indigenous groups in the wards are migrants from other states in the country (examples include the Eguns, Igedes, and Ohoris); as well as migrants from the neighboring Benin Republic—this group are popularly referred to as “the Cotonous.”

Ipara and Ilara had estimated under-5 populations of 1,820 and 1,302, respectively (SPHCDA 2017). We sampled 210 adults representing 215 under-5 children, using the WHO modified two-stage cluster sampling method (13), with a 100% response rate in selected households. A total of 30 clusters were selected from both wards according to their relative populations. To estimate the difference between the proportion of unimmunized children- estimated at 23% (based on NHMIS data) and an endline estimate of 10% (alpha of 5% and power of 80%) would have required a sample size of 127 children. However, we aimed to study a minimum of 210 children (at least 7 children from each of 30 clusters) across the two selected wards. Individuals were eligible as survey respondents if they were caregivers of children under-5 and were currently domiciled in the ward. We aimed to exclude individuals with speech and perceptual challenges based on inability to communicate or respondents showing difficulty with understanding date, period of the year or purpose of the interaction/study but none of such individuals were encountered during the study.

For the FGDs, purposive sampling was employed. A total of 16 FGDs were held with community men and women in the two wards. Adults who were caregivers or involved in the immunization decision-making relating to a child (or grandchild) were included in the discussions. Research assistants recruited participants with the help of community mobilizers. To ensure the free participation of men and women, participants were separated according to gender and age—young women of child bearing age and older women; young men and older men. Investigators ensured a blend of socio-economic groups during the sampling of participants and the FGDs were conducted until saturation of information was achieved.

A total of 25 stakeholders involved in immunization service delivery were recruited for SSIs using purposive sampling and in some cases snowball sampling. They included policy makers, local government implementers, health workers, and community (committee) leaders. We aimed for diversity and interviewed different stakeholders in the various categories. We continued interviewing until no new information emerged and saturation was reached.

### Data Analysis

**Figure 1:**
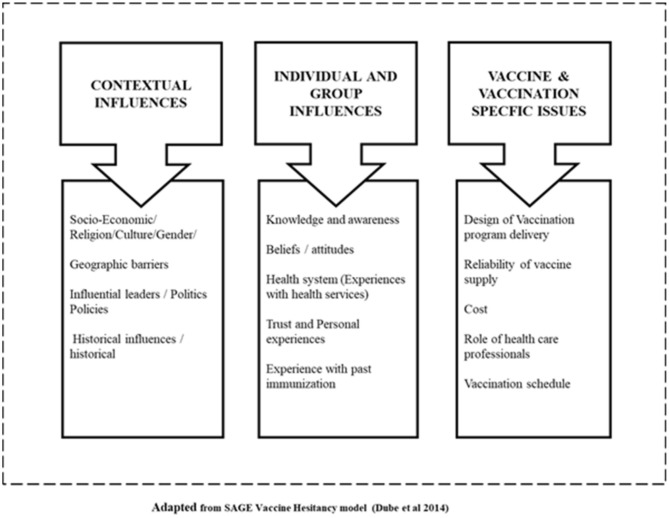
Conceptual framework.

We adapted the WHO Strategic Advisory Group of Experts (SAGE) Vaccine Hesitancy model (14) which mapped the determinants of vaccine hesitancy based on systematic review of literature and interviews with immunization managers of state and national levels immunization programmes in 13 countries (see Figure 1). The model is based on the premise that attitudes to vaccination is a continuum ranging from complete acceptance to total refusal. Vaccine hesitancy is defined as a locus within this continuum and could result in acceptance of some vaccines and refusal of others, delayed vaccination and tentative acceptance, thereby influencing overall immunization utilization. The model differentiates between contextual, individual, group, and vaccine/vaccination-specific factors that influence immunization acceptance and utilization. The causes of vaccine hesitancy were found to be context-specific and Dube et al. (14) noted the need to identify locally relevant factors in order to develop appropriate strategies to tackle them.

Recognizing that the health sector, including immunization services, is a complex adaptive system, and different elements within the context are interlinked, and influence how immunization services function, we took a systems perspective: since the REW strategic components have community participation elements, we also explored the policies on community participation and action. We used the SAGE model in our analysis to group the determinants of vaccine hesitancy and immunization use in both contexts; and to gain more insight into contextual and other influences.

The conceptual framework is presented in Figure 1.

This study was the baseline for a participatory action research on immunization. An aim of the quantitative component was to derive a measure of immunization uptake pre-intervention which would be supported by NHMIS data.

Primary quantitative data was entered into Statistical Package for the Social Sciences version 21^2^ by trained data clerks. A wealth index was derived using productive and non-productive household assets, household amenities and other measures of household living standard. Immunization was assessed as complete if an immunization card was seen and three doses of DPT/Pentavalent vaccine as well as measles and yellow fever had been recorded as administered. A secondary measure of immunization completeness was derived and reported as present for individuals satisfying the primary outcome or reporting ownership of an immunization card (which could not be provided for inspection) and reporting the child had received DPT/Pentavalent, measles and yellow fever vaccines. Descriptive statistics were calculated for variables using a survey design adjusted logistic generalized linear model. As the sample framework used a proportion to population sample to assign clusters within wards and to select clusters within wards, the total sample was considered to be self-weighing at the ward level. Therefore, weighing of individual observations proportional to their respective sampling fractions were not applied.

A multivariate logistic regression was performed for children above the age of 9 months to identify factors associated with completion of immunization. Ward, age and gender of child, employment status and highest level of education of caregiver, and wealth quintile of the household were taken into account in this analysis to adjust for confounding and possible association between these individual factors. Ninety-five percent Confidence Intervals (CIs), lower and upper CIs (LCI and UCI), Odds Ratios (OR), Standard Errors (SE), and *p*-values (p) of the factors included in the model are presented in **Table 4**. Please take note that cautious interpretation of this data is advised as the sample size is relatively small. We therefore limited the number of factors included. A general rule of thumb is that, per factor included, at least 10 cases for every category of the factor should exist.

FGD and SSIs were audio-taped and transcribed. Data was analyzed using the qualitative data analysis software, NVivo 11^3^. An inductive approach and open thematic coding were used. Transcripts were read and coded by two qualitative researchers, using common themes and sub-themes according to the conceptual framework. A third qualitative researcher coded a few transcripts to ratify the codes and themes/sub-themes identified. Analysis was conducted iteratively using a three-pronged approach: “noticing, collecting, and thinking” (15). We aimed to understand immunization utilization in both wards; if there were vulnerable groups; and whether there were differences in opinions and experiences between specific groups and between the two wards.

Triangulation of data was carried out using quantitative and different qualitative methods to ask the same questions, and asking different types of respondents the same questions. This enabled us to identify areas of agreement and disagreement between and within groups of respondents. We compared and contrasted answers between different respondent groups and between the two wards. We assigned weights in the qualitative analysis using the frequency of respondents' perceptions and agreements between different interviews and respondents.

## Results

After the description of respondents, the relevant influencing factors are presented according to the conceptual framework. Where applicable, the survey results are presented before the qualitative findings.

### Background and Respondents' Characteristics

Out of the 210 households surveyed, 124 households (59%) were studied in Ipara- the more populated of the two wards. All the 210 respondents were female caregivers. Most were Yoruba (89%)—this was a reflection of the general population ratio between indigenous people and migrants. Over 90% reported that they had attended school in some form (pre-school, primary, secondary, or higher education). Nevertheless, 32% of the respondents were unable to read the basic sentence provided and were therefore considered functionally illiterate (details in Table 1).

**Table 1:** HHS—respondents' background and characteristics.

**Variables**		**Respondents' background and characteristics (*****N*** **=** 210^*^)	
		**Frequency**	***N*%**
Location	Ilara	86	41.0
	Ipara	124	59.0
Religion	Christianity	173	82.4
	Islam	29	13.8
	Others	8	3.8
Ethnicity^**^	Yoruba	188	89.5
	Others	22	10.5
Employed	Yes	168	80
Highest level of education	None/Pre-school	33	15.7
	Primary	73	34.8
	Secondary	90	42.9
	Higher	14	6.7
Literacy	Cannot read at all	69	32.9
	Able to read only parts of sentence	44	21.0
	Able to read whole sentence	93	44.3
	Other (refused to read or visually impaired)	4	1.9
Age of respondent (Years)	≤20	18	8.6
	21–30	84	40.0
	31–40	79	37.6
	41–50	20	9.5
	≥51	9	4.3

* *All the survey respondents were female caregivers*.

** *Ethnicity relates to indigenes (Yoruba) and migrants (including the Igedes, Eguns, and foreign nationals from Benin Republic referred to as the “Cotonous”)*.

A total of 16 FGD (8 in each ward) were carried out. There were 6–7 respondents in each FGD (*N* = 98) and the characteristics of the respondents are summarized in Table 2.

**Table 2:** Characteristics of FGD respondents.

**Variables**		
Location	Ilara	8 FGD (Young women, Young men, Older men, Older women). Two sessions in each category. Six to seven participants per session
	Ipara	8 FGD (Young women, Young men, Older men, Older women). Two sessions in each category
Age of respondents	Young women/men	19–40 years
	Older women/men	Above 40
Religion	More Christians than Muslims but both religious groups were well-represented. Four traditionalists (2 men and 2 women)	
Occupation	Traders	30 participants (18 women and 12 men)
	Farmers	Mostly men (12 men; 1 woman)
	Hair stylists	All were women (5)
	Tailors	All were women (3)
	Professional drivers	All were men (5)
	Retired (teachers, nurse)	3 (1 man and 2 women)
	Clergymen	All men (6)
	Others (one teacher, students, artisans such as Electricians, painter, welder)	8 [7 men; 1 woman (teacher)]
	Homemakers or no definite occupation given	25 (13 women and 12 men)
Number and age of children of respondents	Young women	Number of children ranged from 1 to 5
		Age range: 3 weeks to 20 years
	Older women	Number of children ranged from 2 to 7
		Age range: 4–45 years old
Marital status	All respondents except one (a widow) were married.	

We interviewed a total of 15 policy makers, local government implementers, and frontline health workers. Their specific functions are displayed in Table 3. Ten community stakeholders were also interviewed—they consisted of religious leaders and the foremost traditional rulers in both wards; and (post-holding) members of three different community mobilization structures. The WDC was not functioning in Ilara, and members of the Community Development Association (CDA)—set up by the community to address general development issues including health—were interviewed instead. Table 3 gives details of the community stakeholders.

**Table 3:** SSI respondents at state, local government, and ward levels.

**Ogun State Policy makers (5)**	**Remo-North Local Government officials (5)**	**Health workers at ward facilities (5)**	**Community stakeholders—Ipara (5)**	**Community stakeholders—Ilara (5)**
Permanent Secretary of the Ministry of Health	Principal Medical Officer of Health (PMOH)	Health Worker In-Charge—Ipara	WDC Chairman	CDA Chairman
State Immunization Officer (SIO)	Local Government Immunization Officer (LIO)	Health Worker 1 (Ward focal person), Ipara	WDC Secretary	CDA member
State Cold Chain Officer	Cold Chain/Logistics Officer (CCO)	Senior Community Health Extension worker (CHEW)—Ipara	Foremost traditional leader—Ipara Baale	Foremost traditional leader—Ilara Kabiyesi
State Health Educator	SMC Chairman	Health Worker In-Charge—Ilara (Ward Focal Person for immunization)	Religious leader— Pastor	Religious leader— Pastor
Zonal Coordinator, National Primary Health Development Agency	SMC Secretary	Health assistant, Ilara	Religious leader— Imam	Religious leader— Imam

### Immunization Utilization in Ipara and Ilara Wards

The mean age and range of the children whose caregivers responded to this study was 24.4 ± 15.7 months, and more than half of them were male. The RI schedule in Nigeria administers the final antigens (measles and yellow fever vaccines) at 9 months of age. The analysis of immunization completeness encompasses all children older than 9 months who should have plausibly achieved this outcome. Only 56 children (32.6%) of the 172 children over 9 months of age had immunization cards available for inspection. 23 (59.6%) of these children were fully immunized, noticeably higher at 67.6% in Ipara when compared to 47.8% in Ilara. However, when immunization status was assessed by card and recall 146 (84.9%) of the 172 children were reported as fully immunized, with 88.1% in Ipara and 79.3% in Ilara (*p* < 0.05). The utilization figures reported by recall were most likely not reliable—caregivers cannot be expected to recall number of immunization doses with precision and this figure may approximate immunization commencement rather than completion.

### Factors Influencing Immunization Use

#### Contextual Influences

Contextual influences on immunization utilization explored related to the socio-economic factors, religion, culture, gender, geographic barriers, politics, and policies.

##### Socio-economic/religion/culture/gender

The reasons given for the levels of utilization of immunization services in the communities included ethnicity, culture, household decision making, and gender relations. There was no major difference in the survey and in the qualitative interviews regarding utilization of immunization between the three main religious groups (Christian, Muslim, and Traditional) or between people of different socio-economic status in both wards. In the survey, though caregivers from households in the 3rd or 4th quintiles were more likely to fully immunize their children compared to those in the other quintiles, this was not statistically significant (see Table 4).

**Table 4:** Multivariate logistic regression.

**Variable**	**Vaccination coverage by card and recall** **(*N* = 172)**				**UV regression results**		
	**Complete immunization**		**95% CI**		***OR***	***SE***	***p***
	**N**	**%**	**LCI**	**UCI**			
**Location**							
Ilara (N = 63)	50	79.4	69.80	89.00	–	–	–
Ipara (N = 109)	96	88.1	82.42	93.78	1.92	1.48	0.108
**Child age group**							
9–11 months (*N* = 8)	7	87.5	64.37	110.63	–	–	–
11–23 months (*N* = 59)	51	86.4	78.76	94.04	0.91	2.76	0.927
24–59 months (*N* = 105)	88	83.8	76.55	91.05	0.74	3.22	0.799
**Child gender**							
Female (*N* = 80)	67	83.7	74.96	92.44	–	–	–
Male (*N* = 92)	79	85.9	78.53	93.27	0.85	1.66	0.750
Parent's highest level of education^*^							
Preschool (*N* = 12)	9	75	47.56	102.44	–	–	–
Primary school (*N* = 61)	54	88.5	79.68	97.32	2.56	2.44	0.300
Secondary school (*N* = 74)	64	86.5	78.66	94.34	2.12	2.36	0.380
Higher (*N* = 8)	8	100	100.00	100.00	1.47	2.25	<0.0001
**Wealth quintile**							
Lowest quintile (*N* = 29)	25	86.2	74.05	98.35	–	–	–
2nd quintile (*N* = 40)	32	80	67.65	92.35	0.64	1.98	0.518
3rd quintile (*N* = 59)	52	89.7	82.64	96.76	1.39	1.73	0.557
4th quintile (*N* = 15)	13	92.9	79.96	100.00	2.08	3.00	0.512
Highest quintile (*N* = 29)	23	79.3	63.62	94.98	0.61	1.97	0.478

* *Parents with no schooling not included*.

*Culture and Ethnicity* played important roles in both Ipara and Ilara. Some cultural factors were illuminated when FGD respondents were asked about which seasons and events made it difficult for them to bring their children for RI. Several traditional festivals were described as events where the women were unable to come due to traditional rituals and imposed curfews. However, the perspectives of women and men differed. The women specified the months from September to December as months that were particularly difficult because of these festivals and events.

“*Oro festival always impedes the immunization exercise because women cannot go out.” (Young woman, Ipara)*

However, this notion was dismissed by the older men who stated that the women were busy for other reasons.

“*What we noticed is that those mothers, once it is the period of washing kola nut or going to the market, they may see spending one hour at the maternity as not being convenient. But all these usually happen between September and December - the period of washing kola nut - that period they are always very busy; but I think spending just one hour on their child in a month should not be too difficult a task.” (Older man, Ipara)*

The indigenous Yorubas were perceived by the health workers, policy makers and FGD respondents as utilizing immunization the most compared with migrant groups. Poor utilization by migrants was perceived as mainly due to their cultural beliefs especially those that valued traditional above western remedies. Additionally, the Cotonous were reported to refuse immunization for their children mainly for reasons associated with lack of trust in the quality of the health services which they considered as inferior to the health services in Benin Republic. Low utilization by this group was said to be further compounded by their preference for home deliveries, language barriers and their occupation (farming)- which made them unavailable for scheduled immunization activities. Nevertheless, among all the groups, the Igedes were frequently mentioned as the most resistant to the immunization of their young children. Some FGD participants in Ilara described them as being intractable in their stance even in the face of threats by the Kabiyesi (king) to eject them from the communities.

“*Some of them (the Igedes) are very stubborn, they won't take the vaccine, no matter what.” (Young woman, Ilara)*

*Household decision making* dynamics illuminated the *gender relations/roles*. The majority of caregivers (all females) in the HHS (88.6%) reported that they were the ones who made the decision to immunize their children. Additionally, 60% of the respondents named themselves as the primary influencer (67.7% in Ipara, statistically significantly more than the 46.7% in Ilara) while 19% named their spouses as influencers. These answers were remarkably different from those from the FGDs. In the FGDs, the women reported playing strong roles in affecting/-and effecting decisions on immunization of their children but the men were the primary decision makers on immunization issues. However, respondents reported numerous influences outside the nuclear family that directed immunization utilization decisions: the men were strongly influenced by their mothers, while the women valued the direction of their fathers/fathers-in-law who also happened to be the elders in the communities. The summary of the effect of this gender interplay is that even if women wanted to immunize their children, they could not do so if their husbands did not agree; or if their husbands' mothers refused. Additionally, the young women listened to the elders which affected their decisions to immunize their children.

##### Geographic barriers

Eighty-one percent of respondents in the survey regarded the distance to immunization facilities as “short or very short.” This was similar to findings in the FGDs where health facilities were reported as being generally within walking distance to many households in both wards. However, a disadvantaged group mentioned especially in the SSIs were people living in “hard-to-reach” areas like Aba James and Ifote, which had difficult terrains and were usually inaccessible during the rainy season.

Local government officials and health workers described low utilization of RI at health facilities in both wards (more in Ilara than Ipara) adding that gains so far recorded in the programme were partly due to outreach activities. However, they noted that geographically disadvantaged areas did not benefit much from outreaches due to financial limitations in the programme.

##### Policies and politics

According to the policy makers and local government officials, there are national and local policies supporting community participation and action in immunization. Immunization is a priority issue in the country, supported by GAVI and other multilateral organizations. There are clear policies related to the immunization programme and structures in place for implementation of the strategies and plans linked to policy. They noted that successful adoption and implementation within the local contexts were reliant on cooperation from the community leaders and community members—with monitoring being key.

The SMC are responsible for immunization campaigns and community mobilization as well as conflict resolution relating to immunization issues in the wards. The WDC acts as partners providing a gateway to the communities and support in community mobilization and outreaches.

Respondents in the survey (58 and 34%, respectively) named the SMC and WDC as the main community structures linked to immunization and important sources of information regarding immunization. In the FGDs, the young women (especially in Ilara) reported being unaware of any committee responsible for immunization or health in the community. The older men and women however described the community committees but those in Ilara noted that the WDC had been defunct in the ward since 2014 due to excessive politicization of the committee which led to the loss of interest of the traditional ruler. According to the respondents, before it became defunct, the WDC used to have monthly meetings with the king and the health workers in the primary health center. The Ilara CDA had therefore been in charge of immunization issues since 2014 but was described in the FGDs as “functioning poorly.” The CDA was described by both the older men and women as being politically motivated, with posts in the committee assigned by the local government in power.

The Ipara WDC was also described as being politically motivated but rated as functioning well by the older men and women groups; and more cautiously by the young women:

“*Well, what I can say is that, they try their best but you know our people, nobody wants to do things for free.” (Young woman, Ipara)*

When asked about their perception of their wards, the majority of the respondents in the FGDs in Ilara stated that Ilara was marginalized within the LGA. This was in contrast to their Ipara counterparts, most of whom were of the view that Ipara was a progressive ward.

#### Individual and Group Influences

The main factors related to individual and group influences were knowledge and awareness of the value of immunization, beliefs and attitudes toward immunization, past experiences with immunization and health services factors which influenced trust and personal experiences of caregivers and household decision-makers.

##### Knowledge and awareness

The only statistically significant factor (note low cell frequencies in some instances) of complete immunization status for children above 9 months was the completion of higher education by the mother (*OR* = 1.47, *p* < 0.0001) (see Table 4).

The study showed evidence of awareness and knowledge of the value of immunization in both wards—more in Ipara than Ilara—with reported need for more awareness raising and knowledge improvement in several areas like the need for completion of immunization and understanding of vaccine schedules. In the survey, majority (95.7%) of the respondents (99.2 and 89.6% in Ipara and Ilara, respectively, *p* > 0.05) stated that immunization prevents diseases, with polio and measles being the vaccine preventable diseases that they were most aware of. However, only 37.7% of respondents [Ipara (45.9%) and Ilara (23.4%), *p* > 0.05] knew when the dosages of the different immunizations should be given. The importance of all doses was recognized by 88.1% of respondents, more so in Ipara (95.5%) than in Ilara (75.3%)—statistically significant at a 5% level of significance determined by non-overlapping 95% CI.

The findings in the FGDs and SSIs confirmed that there was a “good” level of awareness and knowledge about immunization and its value. However, most policy makers and local government officials responded that there was a need to improve awareness and knowledge for all the groups. Some described an issue whereby some care givers would think their children had completed vaccinations by taking only one vaccination. Traditional and religious leaders in both wards noted that awareness and knowledge were hampered by insufficient health education:

“*All tribes and religions support it (immunization) because no one wants to die. It is because the publicity of this program is not enough in our community that causes the low turnout.” (Community leader, Ipara)*

There were differences between the key sources of information relating to immunization and caregivers (mostly mothers)' information-seeking behavior. Health workers were reported in the survey (85.2%) as the most important (and commonest) source of information about immunization. This finding was supported by the FGDs. In Ipara, statistically significantly more respondents (92.5%) than in Ilara (72.7%) indicated health workers as the commonest source. However, according to the FGDs, young women appeared to resort first to the elders (older men) in the communities for answers to their immunization questions or to the members of the WDC and CDA, who then would point them to the health workers. This finding was interesting since many of the young women could not mention the names of the WDC/CDA members. It is possible that the young women valued the opinion of the elders because of their standing within the family structure rather than because of their roles in the community structures.

##### Beliefs/attitudes

Common beliefs in the communities about immunization which were mentioned by the caregivers in the FGDs include beliefs that immunization: kills children; was the “white man's” way of achieving family planning and population control; causes deformities in children especially when given to the pregnant mother; and could actually cause paralysis in children. Many respondents (especially the older women and men) in the FGDs said that they did not believe this anymore having seen the benefits of immunization. However, some of them expressed that the traditional ways could not be discounted completely, that the elders knew how to treat certain diseases like measles better than the protection offered by the vaccines. Indeed, the issue of measles came up frequently in the interviews as an area of tension between orthodox and traditional medicine. In both Ilara and Ipara, vaccine hesitancy was most frequently reported for measles vaccine compared to the other vaccines on the immunization schedule.

##### Health services factors

Key health services issues mentioned by respondents include the absence of antenatal and delivery services in Ilara; lack of well-equipped and functioning health facilities; and shortage of health workers.

The *presence of antenatal and delivery services in the health centers* played a key role in driving immunization utilization. This was seen clearly in Ilara where FGD respondents reported that the absence of delivery facilities discouraged women of all tribal groups from using immunization services at the facility. In Ipara, delivery services at the health facility promoted the use of health and immunization services. Young mothers in Ilara expressed the need for a health center where they could have antenatal services but noted that the current health center needed to be more functional to meet those expectations. Young mothers in Ipara were happy with delivery services in the health center but wanted the facility to be upgraded to also take deliveries for primigravid women so as to improve immunization use by that group of stakeholders. However, young women in the FGDs in both wards stated that the immunization outreach services in markets, schools, churches and mosques were very useful in ensuring that people that would not come to the health centers to get their children vaccinated were reached.

Community members' perceptions about *conditions of the health facilities*, were mostly unfavorable especially in terms of the environment, poor/inadequate infrastructure and lack of equipment and supplies, more so in Ilara than Ipara. According to the FGDs, this resulted in a reluctance of the community members to access care in their health facilities further reducing RI utilization. This was supported by the survey-−55% of the respondents reported that the last immunization taken for their child(ren) was from the fixed government health facility while 34% reported outreaches as their source.

“*If you don't have money, you can explain to the health worker politely and they will understand, but if you say it in an aggressive way like saying, ‘we learnt it is free why are you collecting money?' it is not good. Although they will still give the immunization because it is free, after they have given immunization, they will request for a token.” (Young woman, Ipara)*

Regarding the availability of health workers to carry out immunization, the respondent groups were unanimous in their answers that there was a shortage of health workers and described that as an important issue linked to the availability of vaccines.

##### Experience with past immunization

There was consensus in the FGDs in both wards that Adverse Events Following Immunization (AEFI) were the greatest demotivating factor against completion of immunization. AEFI also promoted fear among young mothers and fathers, thereby deterring initial use of immunization. It was also the reason for reported loss of confidence by the community members (especially young men in both wards) in the quality of vaccines; and loss of trust in the competence of the health workers. Additionally, the distress caused by the excessive crying of the children due to fever and swollen limbs was reported as the reason why some of the young men instructed their wives to discontinue immunization of their children.

“*What I also think is that, sometimes the swollen arm might not be caused by the vaccine but by the person who administered it. He might be too hard in injecting the patient or giving it in the wrong place. It has happened to the people we know; it has even happened particularly to my wife. Her arm was swollen” [Another respondent interrupts] “When my baby took the injection, his arm was swollen and he was weak and I was wondering if this will encourage immunization, these things caused me to become skeptical about immunization.” (Young men, Ipara)*

Nonetheless, many of the community stakeholders in the SSIs in both wards were of the opinion that overall immunization was generally well-utilized and that this was evidenced by the reduction in childhood diseases and mortality.

#### Vaccination Services Specific Influences

Key findings in this section relate to reliability of vaccine supply; costs; and role of health care professionals.

##### Reliability of vaccine supply

Unavailability of vaccines at the scheduled times was the most frequent complaint by the FGD respondents in both wards. Young mothers in both wards expected that in addition to decreased waiting times, vaccines should be regularly available in health facilities and administered according to the immunization programme schedule. Logistical challenges resulting in vaccines being largely unavailable on schedule for routine immunization were acknowledged by the health workers and policy makers though many emphasized the availability of vaccines at the local government level. One major reason given for the problem was the need to transport vaccines from the cold store in the local government headquarters (Isara) on RI days. Furthermore, recipients had to reach a critical mass (estimated range from 9 to 20 children) before some vaccine vials could be opened for use. Inadequate electrical power supply further challenged the vaccine cold chain and thwarted the possibility to store vaccines at facilities.

##### Cost

Sixty percent of the survey respondents reported that there was no direct or indirect cost for immunization; 29% considered the cost of the service as cheap and 4% thought it was expensive. Unavailability of vaccines at the scheduled times in the health facilities contributed to indirect costs of immunization. To overcome the logistical challenges, respondents described contributing money for the transportation of the vaccines. There was consensus among the FGDs participants that the money paid for the transportation of the vaccines was not really the problem—they were more concerned about the availability of the vaccines according to the schedule, which did not seem to be assured regardless of payments made. Nonetheless, many of the FGD respondents complained about the money used to pay for immunization cards, exercise books, pens and occasionally syringes and needles. Additionally, in Ipara, some of the young women referred to giving “tokens” to health workers.

“*If you don't have money, you can explain to the health worker politely and they will understand, but if you say it in an aggressive way like saying, “we learnt it is free why are you collecting money?” it is not good. Although they will still give the immunization because it is free, after they have given immunization, they will request for a token. (Young women, Ipara)*

##### Role of health care professionals

Though in the survey, 89% of respondents reported health workers' behavior as “helpful” or “very helpful,” responsiveness of the health workers in relation to vaccination services was considered unsatisfactory by the FGD respondents from both wards. The FGDs provided a platform for more detailed assessment of health worker behavior: respondents complained about health workers not sending reminders on time about RI or outreach days, and blamed them for AEFI such as swollen injection sites. Complaints of unavailability of vaccines according to schedule and the resultant long waiting times were attributed to health workers' ineffectiveness.

However, health workers, local government officials and policy makers rated the current immunization programme as responsive to the needs of the communities in Remo North LGA—with trained and capable health workers, though seriously short-staffed. All reported widespread staffing shortage for immunization activities resulting in heavy workload for available staff, further exacerbated by additional assignments from other programmes.

“*…The Community Health Extension Worker on duty at a time, will be the one to vaccinate the children, and also attend to patients, the work load is much for us…” (Health worker)*

## Discussion

Remo North was highlighted as the LGA with the highest number of unimmunized children in Ogun state. Though the majority of caregivers in the survey reported that they had completely immunized their children, this could not be validated as the vaccination cards for many of the children were not available to be assessed. Estimation of immunization coverage by maternal recall, though an accepted practice in developing and developed countries (16, 17), is fraught with the likelihood of recall bias. The assessment by cards only, highlighted low immunization coverage (59.6%)—this is likely a more reliable picture despite the small sample size. Similar low immunization coverage has been seen in other studies in Nigeria (5, 18–20).

The study identified many determinants of immunization use. However, it is important to identify the key drivers in order to design credible and realistic interventions.

Contextual factors driving immunization utilization in both wards were mostly alike but there were a few important differences. The *socio-economic/cultural and gender* factors elicited in this study showed different structures in the wards which promoted inequalities in immunization use. Cultural beliefs (such as traditional methods being better for dealing with childhood diseases especially measles); rumors; and the politicization of community links to immunization delivery clearly (negatively) affected immunization utilization in this study—more in Ilara than in Ipara. The migrants in both wards also did not utilize immunization well. However, the frequency with which this was mentioned by all groups of stakeholders may also be due to some level of ethnic-based bias. It is to be noted that these groups were a minority (consisting only about a tenth of the population) and therefore could not account for the magnitude of the issues especially the low coverage in Ilara which clearly also suggests non-utilization by indigenous people. Involving traditional leaders in the communities in the immunization programme was seen as important in this study due to their level of influence. Nevertheless, this has not tackled the problem of poor utilization of immunization by indigenes and migrants in both wards. Nonetheless, immunization coverage in Ilara declined precipitously from 2014 to 2015 coinciding with the demise of the WDC in 2014 and this overlap in timing suggests that the WDC backed by the foremost traditional ruler was a major driver of immunization in that ward. Though not much is documented in Nigeria, this finding is supported by Sagar et al. (21) who noted that in India dropouts resulted from poor community linkages.

Many of the contextual factors identified in this study cannot be easily addressed since they are entrenched in the cultural and political strata and may be outside the influence of the health sector. However, the social mobilization structures (WDC and SMC) are already embedded in the design of the vaccination programme and strategies can be developed to strengthen these (and minimize political interferences), in order to drive the social and behavioral change needed to overcome vaccine hesitancy and improve immunization utilization.

Though the study showed that young women in Ilara and Ipara were knowledgeable and aware of immunization and its value, the poor knowledge of vaccine schedules and times for the doses displayed by Ilara young women gave a hint as to why their understanding of the value of immunization had not translated to more utilization. Gender patterns in decision making and the more generalized dissatisfaction expressed in Ilara household and community networks of influence regarding their health services may also account for this. However, the gender differences displayed in the perceptions relating to barriers to access that women experienced during festivals suggests that there may be a need to increase awareness among males in order to tackle this hindrance.

There were contradictions in the views of young women in the survey and all the stakeholders in the qualitative interviews regarding who was the primary influence in immunization at household level. Policy makers, health workers and many community members in the IDIs and FGDs were of the view that the final decision is with the husbands. It is possible that this question was not asked in a way that was understood by the respondents in the survey. Some of the caregivers may have regarded the fact that they were the ones who took the children to the health center for immunization as equating to primary decision making.

*Health service factors* were key drivers of immunization utilization in this study. Health workforce shortage was a frequently mentioned problem which hindered immunization service delivery in both wards—though to a lesser extent in the semi-rural Ipara which is more likely to attract and retain health workers than Ilara. Health facility (institutional) births also improved the likelihood of immunization utilization in Ipara where antenatal care services in the health center provided a portal for health/immunization education and awareness. Similar studies in Nigeria (22, 23), Ethiopia (24), and other contexts (21, 25) have documented that health facility births positively influence immunization use and completion.

AEFI was perceived by the respondents as the major cause of loss of confidence in the competence of the health workers and the quality of the vaccines in both wards. This issue of loss of trust in immunization services has important consequences and has led to the boycott of polio vaccination in some parts of Nigeria in the past (9, 26). The fear of AEFI contibuted to vaccine hesitancy, low utilization of immunization and dropouts in both Ilara and Ipara—a finding supported by studies in Nigeria (27, 28) and other contexts (29).

An important supply-side limitation common to both wards was the irregularities associated with the availability of vaccines for RI in the health facilities—a common finding in other Nigerian studies (22, 23, 27), and usually as a result of logistical problems rather than stock out. Unavailability of immunization cards was also an important problem and has been documented extensively (24, 28, 30–32), with many studies showing that availability of immunization cards improves the likelihood of children getting immunized. Evidence clearly shows that when immunization cards are readily available and proper information recorded in them, this enhances continued use of immunization by community members (28, 33, 34). Additionally, this would improve validity and enable savings from not revaccinating children needlessly (35).

Overall vaccine hesitancy was exhibited more in Ilara than Ipara. The SAGE model has helped us to group the determinants of vaccine hesitancy and immunization use in both contexts better. However, because health (and immunization) services is a complex adaptive system, it is difficult to fit everything into the three linear boxes in the framework as the factors are interlinked. For instance, trust and personal experiences with immunization are influenced by both health and vaccination services factors and can also be affected by considerations that are culturally driven.

Furthermore, though Ilara and Ipara are both in the same LGA, their (different) contexts play critical roles in the success (or failure) of the vaccination programme. This displays a need for locally context-specific strategies and approaches to addressing the issues related to vaccine hesitancy and immunization utilization.

Low immunization coverage (26%) in Ilara points to a need for critical, swift and practical solutions—key among which is the improvement of awareness and knowledge of the vaccination schedule. Improving the condition of the Ilara health facility and provision of antenatal and delivery services to the young women would encourage the utilization of health (and immunization) services. Reviving the WDC in Ilara is also important to ensure effective community mobilization and drive demand for services.

There is an urgent need to strengthen capacity for vaccine pharmacovigilance in both Ilara and Ipara in order to recognize and respond speedily to adverse events. Likewise, it is necessary to address reasons associated with dropouts; and an important place to start would be to ensure that vaccines are available at scheduled times without costs to the users. Also, though their proportion is small, it may be useful to tackle poor utilization by migrants in order to achieve full immunization coverage.

### Limitations of the Study

Due to the need to understand perspectives on immunization among mothers of under-5 children broadly, we did not limit the immunization completeness assessments to children aged 11–23 months in the survey. Consequently, the precision of estimates of immunization coverage was compromised. We expect that the increased scope of understanding across the broader age group compensated for the loss of immunization completeness precision. Modeling variations in immunization utilization across different socio-economic and demographic factors were greatly constrained by the relatively small sample size of the survey. This limited the power to detect statistically significant differences in vaccination use resulting in the mostly descriptive nature of reported findings.

There is a likelihood of recall bias, especially since children under 5 years of age were taken into consideration. Caregivers cannot be expected to recall number of immunization doses with precision and figures given may approximate immunization commencement rather than completion.

We did not collect quantitative data on AEFI—this could have added more value to the study.

Recruitment of the respondents for the FGDs was carried out by the research assistants in consultation with community stakeholders. Though confidentiality and privacy were assured, social desirability, and recall bias regarding immunization utilization in the wards cannot be ruled out completely.

## Conclusions

Immunization utilization and coverage in Remo North is driven by interlinked community and health services issues—a finding characteristic of this complex adaptive system—and points to the importance of actors at both supply and demand levels to be involved in the immunization service delivery and interventions. Intervention approaches should ensure that community priorities are addressed and strategies adjusted to suit contexts.

## Data Availability Statement

The data that support the findings of this study are available from the Royal Tropical Institute, The Netherlands; the Ogun State Government in Nigeria and the grant funders. The data are available on request.

## Ethics Statement

Ethical approval was obtained from the University of Ibadan, University College Hospital Ethics Board (UI/UCH Ethics Committee assigned number UI/EC/15/0447). Informed consent was obtained from all the study participants. Privacy and confidentiality was assured for all respondents. Additionally, permission for the study was granted at three levels—the state Primary Health Care Development Board/Ministry of Health, the Remo North LGA and from the Community through established Community Development Associations.

## Author Contributions

NA and MD contributed substantially to the study design, data analysis, interpretation of findings, drafting of the manuscript, and critical revision of drafts. JB contributed substantially to the interpretation of findings and critical revision of drafts. NA, EO, and OP were involved in the field work and data collection. EO, OP, and AA contributed to data analysis and drafting of the manuscript. All the authors approved the final manuscript.

### Conflict of Interest

The authors declare that the research was conducted in the absence of any commercial or financial relationships that could be construed as a potential conflict of interest.

## Footnotes

^1^This percentage is because of target population issues due to faulty denominator – the last national census was in 2006.

^2^© Copyright IBM Corporation 1989, 2012.

^3^© QSR International Pty Ltd.
